# Experiences of mental health problems vulnerability, psychological symptoms and coping mechanisms of displaced adolescents in North-east Nigeria

**DOI:** 10.4314/ahs.v23i1.36

**Published:** 2023-03

**Authors:** Onyekachi Prince David, Jesper Dammeyer, Jonathan Musa Dangana

**Affiliations:** 1 Department of Psychology, University of Copenhagen, 2A Øster Farimagsgade, DK-1353 Copenhagen K, Denmark; 2 Department of Public Health, Babcock University, Ilishan Remo, Ogun State, Nigeria

**Keywords:** Internally displaced adolescents, mental health, psychological symptoms, coping, Boko-Haram

## Abstract

**Background:**

At a global level the issue of forcible internal displacement appears to be somewhat growing. Most internally displaced persons in Nigeria are children and adolescents, but most of the existing literature and public debate focus on the experiences and outcomes of displaced adults.

**Objective:**

We set out to explore the adverse conditions that increase vulnerability to mental health problems, and the psychological symptoms and coping mechanisms reported by internally displaced adolescents.

**Methods:**

Using a qualitative approach, 15 adolescents recruited across three different displacement settlements were interviewed individually using a semi-structured interview format on their displacement experiences, and their adaptations to these experiences.

**Results:**

The thematic analysis showed that, in addition to experience of mental health problems, vulnerabilities and profound psychological symptoms for some, displacement constituted a complex life-changing process for all. High-risk coping strategies such as ‘seeking support by begging’ and ‘transactional sex for exchange of need were predominantly adopted by female participants. Some participants demonstrated resilience by engaging in religious behavioural coping, which they considered necessary to mitigate their past traumatic memories.

**Conclusion:**

Internally displaced adolescents' experience of psychological symptoms and choice of coping mechanisms relate to their contextually perceived needs. Psychosocial support for these adolescents is recommended.

## Introduction

Globally, the issue of internally displaced people (IDPs) seems to be growing rather than diminishing. According to the United Nations High Commissioner for Refugees (UNHCR) 1 report, 45.7 million people are internally displaced due to civil war and international conflicts, famine, and natural disasters. Of most concern, about 15,000 people flee from conflict every day in African countries 2. Currently, there are about 3.3 million IDPs in Nigeria^3^, ^4^. Children and adolescents constitute nearly 60% of IDAs in Nigeria 4, 1.

Since 2009, over three-quarters of internal displacement in Nigeria has been attributed to the protracted Boko-Haram insurgency within the North-east region 5. This insurgency has left many people with no choice other than to stay in designated IDP camps 6. Despite this, children and adolescents living in the camps are exposed to avoidable suffering, in all areas of life, which they have to endure. For instance, in Ethiopia, over 90% of IDAs reported loss of property, 70% suffered extreme thirst, and more than a third had witnessed death 7. Trauma from forced displacement, such as loss of loved ones and loss of livelihoods, has been linked with persistent mental health problems (such as post-traumatic stress disorder)^8^–^12^.

Too few studies examine the contextual mental health problem vulnerability of IDAs, especially those living outside camp settings in Nigeria. These IDAs receive less support because they are less visible compared to IDAs residing in camps. Therefore, understanding IDA's mental health problems vulnerability across different settings in Nigeria, required exploring mental health symptoms from the IDA's own experience.

Coping plays a vital role in determining adjustment to new life situations^13^. None the less, a study among Zimbabwean adolescents showed that a great number of the adolescents used emotion-focused strategies rather than problem-focused strategies^14^. In other studies, adolescents communicated blame or anger towards others.^15^ Social supports seeking 16 and withdrawing from others^17^ have also been reported. These studies have described some types of coping strategies used, but there is a need for further elucidation of coping by exploring the processes and contextual factors involved in coping, especially why a strategy was chosen in a given context. This study aimed to investigate the lived experiences on vulnerability to mental health problems, the psychological symptoms and coping mechanisms experienced by IDAs and the factors underlying their adaptation of such coping mechanisms. This was achieved through a qualitative investigation.

## Methods

This was a phenomenological qualitative study, which explored the experiences of IDAs. It analysed the lived experiences of the participants within their specific context^18^. In addition, the study analysed how participants themselves made sense of their experiences.

### Participants

A purposive sample of 15 IDAs was selected from three different sites: the host community (Jos metropolitan area), unofficial camp (New Kachingoro), and the official camp (Stefano's Foundation). The ability to be interviewed in English were used as criteria in recruiting potential participants. Participants were 9 females and 6 males aged 14-21 years old. Six had been displaced once, 5 twice, while 4 were displaced three times. Other socio-demographic and displacement factors are shown in Table 1.

**Table 1:** Socio-demographic and displacement characteristics of the participants (*N* =15)

Characteristics	N	%
**Gender**		
Male	7	47
Female	8	53
**Marital status**		
Single	14	93
Married	1	7
**Educational achievement**		
Primary	8	53
Secondary	6	40
University	1	7
**Occupational status**		
Unemployed	13	87
Self-employed	2	13
**Duration of displacement**		
1–2 years	3	20
3–4 years	8	53
5 or more years	4	27
**How many time displaced**		
Once	6	40
Twice	5	33
Three times	4	27
**Living with parents**		
Both parents	4	27
One parent	3	20
Relatives/Others	8	53
**Parents Alive**		
Both parents	4	27
Only Father	7	46
Only Mother	4	27
**Special needs**		
School fees	4	27
Food and house rent	5	33
Peace	6	40

### Life situation across IDPs location

National Emergency Management Agency (NEMA) supports IDPs residing in official camps; such camps setting is organized, with full presence of security personal. IDPs in unofficial camps occupy buildings or land they do not own, live in personal constructed makeshift shelter or sometimes in dilapidated and abandoned buildings. In comparison, IDPs in the host community rents apartment share room or lives with a host family or relatives and often rely on them for food 19.

### Ethical issues

Beside the ethical approval obtained from Babcock University (BUHREC581/20) permission were also sorted from the camp manager of Stefano's Foundation IDP camp before the data was collected. Consent was gained from IDAs' above 18 years of age while those under the age of 18 were asked to give verbal assent next to consent by parents or legal guardian. Participants were well informed that their participation was not obligatory, as they were free to withdraw at any time if they chose to.

### Data collection

Data were collected in January 2018. A semi-structured interview guide was used to explore the experiences of IDAs in an open-ended questions format. The interview guide consisted of questions about the experiences that characterised traumatic events, mental health symptoms and coping strategies. Some of the interview questions participants were asked included: ‘what are the experiences you encountered while fleeing from the *Boko Haram-*insurgents? ‘What ways are you affected by those experiences? ‘Describe how you are disturbed at the moment’? ‘What are the resources which you use in order to cope with since you were displaced’? ‘And why did you choose such coping strategies? All interviews were audiotape-recorded and transcribed verbatim. The duration of the interviews lasted between 45-60 minutes.

### Data analysis

Data was analysed using Thematic Analysis. This approach seeks to identify, analyse, organise, describe, and report themes found within a data set 20. The NVivo 11 software was used to enable coding and arrange emergent themes and sub-themes. The analysis was carried out by the first author, and throughout the analysis, emergent themes and categories were discussed with the second and third author. This approach ensured that reduction confirms the data and appropriate bracketing were maintained^21^, as to ascertain trustworthiness 22. The analysis produced three categories, which were mental health problems vulnerability, armed conflict experience and their effects, and coping mechanisms. Key sub-themes and themes emerged from the data are listed in Table 2.

**Table 2:** Major categories, themes, and sub-themes emerging from thematic analysis

Major category	Themes	Sub-themes
**Mental health** **problems** **vulnerability**	Socio-economic and uncertainty	Poor living standard
		Detachment from family and friends
		Uncertainty about the future
**Armed conflict** **experience and** **their effects**	Traumatic events	Staying in the forest and mountains without food and water
		Loss of family members and property
		Witnessing the death of killed victims
		Raped or sexually harassed
	Psychological symptoms	Sadness, worries, and angry
		Uncontrollable thought and sleep difficulties
		Flashback and nightmare
**Coping** **mechanism**	High-risk survival strategies	Seeking support by begging and transactional sex for exchange of needs
	Religious activities and connection	Engaging in prayer, singing religious songs, and attending church
	Meaning-making coping strategies	Religious beliefs

## Results

### Mental health problems vulnerability

Mental health problems vulnerability was the first among the three major topics identified and its themes consists of issues related to social, economic, and uncertainty. All sub-themes are listed in Table 2.

### Socio-economic and uncertainty

'Poor living condition’ was the most prevalent sub-themes within the socio-economic and uncertainty theme, reported by 15(100%) of participants. Poor living condition refer to general lack of accessible resources and deplorable poor living standard - including inadequate health care, poor sanitation, lack of access to electricity and potable water, and irregular mealtimes, among others. The participants recounted the extent to which their current living condition was marked by inadequate resources compared to their place of origin. These excerpts demonstrate this: We are 14 in number living in one small room [...] it makes me unhappy, back home I sleep very comfortable [...] we sleep on mats; I do not even breathe fine (17 Years Old Male, Official Camp).

Seven of us are staying in one room in this place [...] there is not enough water and there is not even electricity [...] we don't have medication, food, and no school for me to attend in this place [...] this environment is very dirty, urine and faeces are all over the corners, no toilet here (16 Years Old Male, Unofficial Camp).

Myself, my mother, and two of my younger sisters are staying in a rented accommodation, it is just a room [...] the stress is much for us here than when we were in our state. It is very hard to even eat sometime; we struggle to eat twice in a day (18 Years Old Female, Host Community).

The sub-theme ‘detachment from family’ was reported by 10(67%) of the participants. It refers to the situation whereby the participants became unable to access their own immediate family members and relatives. Participants' expression showed that displacement disconnects their social life, their friends, and family members. Their responses buttered this:

This crisis separated me from my family members, and this has affected me [...] I no longer see my parents, sibling, and friends (18 Years Old Male, Official Camp).

My father is not here, he stays in Cameroon. After we flee to Cameroon he decided to stay there [...] My brother is taken away by the Boko Haram, you see none of my family member is now close to me (18 Years Old Female unofficial camp)

While we were in our place of origin I had many friends, my relatives were very close to us, we had good time sharing and living well but the Boko Haram has separated us from being together and even in this place I don't have friends (14 Years Old Female, Host Community).

The sub-theme ‘uncertainty about the future’ was recounted by 7(47%) of the participants. They perceived financial difficulties to pursue their education should they continue to remain in their current situation. These excerpts capture this:

This present condition has caused me not going to school [...] I foresee difficulty in funding my education [...] I do not even sleep well because of how I feel about my future (18 Years Old Male, Host Community).

I have finished secondary school and I don't have any work to do at the moment, but I want to continue in the university. My problem now is I am worried because I don´t have the money to register for the Jamb exam (17 Years Old Female, Official Camp).

### Armed conflict experience and their effects

The second prominent category that emerged from the interviews was ‘armed conflict experience and their effects’. Responses were organised into two major themes: traumatic events and psychological symptoms, each theme comprised a number of sub-themes (see Table 2).

### Traumatic events

The traumatic event's sub-theme of ‘staying in the forest and mountains without food and water’ was reported by most of the participants 12(80%). It consists of the difficulties participants endured at various forests they had fled to, such as, starving, sleeping in open space without protection, and the fear of wildlife attack. The participant's responses affirmed this:

I ran to the bush when Boko-Haram came to my village, I stayed in the bush for a week. Nothing to eat. I felt so sad and was afraid of being killed by wild animals (18 Years Old Female, Unofficial Camp).

They were shooting everywhere in the village, and we ran to the bush [...] We stayed three days in the bush, and I was like let God just take my life because I have not slept in the bush in my life. There was a moment I was so much afraid and full of uncertainty (21 Years Old Male, Official Camp).

Another distressing traumatic event for the participants was the ‘loss of family members and property’. Twelve of the15 participants had experienced the loss of a family member and property. These citations captured this:

I have been thinking about the death of my father and how my parents' house and properties were burnt down. (19 Years Old Male, Host Community).

We are two in number, but I am the only child alive as Boko Haram took my brother away, we do not know if he is still alive or not. (16 Years Old Female, Unofficial Camp).

‘Witnessing the death of killed victims’ was one of the traumatic events sub-themes. Nearly half of the participants 7(47%) had experienced such trauma. This refers to various ways participants witnessed how Boko-Haram terrorists killed their family members. This is evident in these excerpts:

Boko-Haram [terrorist] killed my aunt's husband and three other men within our neighbourhood. They were brought in our front and shot dead (21 Years Old Female, Host Community).

Boko-Haram broke our door and forced themselves inside the room [...] they took two of my senior brothers outside and slaughter both of them in front of me and my mum. They later took us inside the room and pointed their guns on us that if we move that they will shoot us (17 Years Old Female, Official Camp).

Fewer numbers of the participants 4(27 %) reported the sub-theme of ‘rape and sexual harassment’. This was a major issue for the female participants. In some cases, men exploited them under the guise of expressing love. These excerpts reveal this:

I have been abused by people [...] there was a day when I was alone in a salon where I sometime go to help the owner, a man walked into the salon, he said that he loves me and I told him sorry I am not interested [...] He started touching my body I had to scream before he stopped (18 Years Old Female, Host Community).

I have been sexually abused by a man who pretended that he loves me here [...] It is not long time, just recent, but I can´t remember the day it happened last but it is like four times now I have been sexually abused (17 Years Old Female, Unofficial Camp).

### Psychological symptoms

The ‘thematic psychological symptoms' refers to participants' continued re-experiencing of the attack of Boko Haram. Responses were grouped into three sub-themes: (a) sadness, worries, and anger (b) uncontrollable thoughts and sleep difficulties and (c) flashback and nightmares. Nine of the 15 participants felt sad, worried, and irritation due to lack of support in the areas they needed help. They were concerned about the harsh condition of their parents who they usually depend on, but now currently depending on others. This worried, frustrated, and depressed them. These excerpts below explain this.

I am disturbed seeing my mother jobless it makes me to be angry [...] everyone here is looking for help from people to survive [...] Since four months now I have been going to some shops to see if I can be employed as a sales girl. They said no they don't want anybody I am sad seeing myself going through all these (17 Years Old Female, Unofficial Camp).

No good thing has happened to me since we flee from our village to this place [...] Life has been worst for us and remembering how Boko Haram destroyed our house and seeing the way my parents are now depending on people's help to survive I feel very sad (14 Years Old Female, Host Community)

Over two-thirds of the participants 11(73%) experienced 'uncontrollable thoughts and sleep difficulty’. The uncontrollable thoughts and sleep difficulty refers to how participants worried concerning the death of their loved ones who provided their needs. Most impacting factor was traumatic exposures followed by their current hardship, which exacerbated their sleep difficulties. These excerpts below capture this:

Oftentimes I do not sleep fine because of how I am suffering now. It worries me [...] each time when I remember how my aunt's husband was killed, who always provided all our needs; it becomes difficulty to fall asleep (21 Years Old Female, Host Community).

I think about how Boko Haram destroyed my village, and this disturbs me so much in my sleep [...] My problem now is that I can be thinking about all that has happened to me every day and I find it difficult to sleep (19 Years Old Female, Official Camp).

Almost all participants 14(93%) reported ‘flashback and nightmare’. Their descriptions showed that they constantly re-experienced the negative effects of Boko-Haram attacks, as they were unable to control their thoughts regarding how their family members were murdered. For some, remembering of those experiences interfered with their daily functioning and this frustrated them. These extracts below represent this view:

In my sleep I dreamt about the Boko-Haram chasing us [...] Sometimes, the experience of how my family members was killed flashes my mind [...] The memory of Boko Haram come to my mind when I see other people living a good life (18 Years Old Male Official camp).

Whenever I remember about the death of my father, I'm not able to concentrate and this frustrates me a lot [...] It comes like nightmare too in my dreams [...] sometimes when they flash the scene of the conflict on TV, I will start to ponder over it [...] Two days ago I had a dream about how my father was killed by Boko Haram (18 Years Male, Host Community).

Often time I remember my cousin that Boko Haram killed [...] Yesterday in my dream I saw how we were running away from our homes when Boko Haram attacked us. When I woke up, I was thinking about it all day (20 Years Old Female, Unofficial Camp).

### Coping mechanism

This category focused on various survival means and coping mechanisms participants engaged in adjusting their situations. Responses were grouped into three themes: ‘resorting to high-risk survival strategies’, ‘religious activities and connection’ and ‘meaning-making coping strategies. All emergent sub-themes are presented in Table 2.

### High-risk survival strategies

The thematic ‘high-risk survival strategies’ were reported by 6(40%) of the participants. They comprised mainly of females in the unofficial camp and the host community. High-risk survival strategies refer to the ways they sought help to proffer solution in areas they were in need. Their responses were grouped into one key sub-theme, ‘seeking support by begging and transactional sex for exchange of need’. Some seek support by going into begging while others seek assistance from people through exchange of sex. Below are some excerpts that buttress this:

Always I leave the camp to beg for food and money [...] some men would not want to help, if you refuse their request [sexual intercourse]. It is a bad experience what will I do. I have to continue begging to survive (17 Years Old Female, Unofficial Camp).

We live with friends [...] they involve me into prostitution so that I can make money, because I need money, I have no other option but to sleep with men (18 Years Old Female, Host Community).

There is a girl who was also displaced from my state [...] she wanted to register for WAEC exam but had no money to pay for the registration [...] A guy asked her for sex and that he would pay for her exam registration if she agreed so she went to the boy's house and after sleeping with her the boy refused to give her the money as promised. She was impregnated by the boy but unfortunately for her the boy denied being responsible and she is now having the child and cannot register for the exam (21 Years Old Female, Host Community).

### Religious activities and connection

The ‘thematic religious activities and connection’, refers to the activities that boost participants inner strength in handling the traumatic experiences and their continual hardship. One potent sub-theme emerged from the theme religious activities and connection, is termed ‘engaging in prayer, singing religious songs and attending church’. Reponses indicated that 11 of the 15 participants used religious activity coping. They turned for such religious activities, which they found helpful in adjusting the impact of the Boko-Haram experiences especially when they feel bad about their condition. The descriptions below demonstrate these experiences:

If the fear of Boko Haram grasps me, I listen to Christian music, read my Bible, and pray to get rid of the thoughts; afterwards I will sense inner peace and feel hopeful [...] believing that soon things will be fine (17 Years Old Male, Official Camp).

When I remember the Boko Haram situation it becomes difficult to sleep fine but when I put everything in my prayer before I sleep, I don't get disturbed in my sleep [...] Sometime I use to pray to God about the way I am feeling, I will just have it off my mind and I thank God for helping me so far in this situation (16 Years Old Male, Unofficial Camp).

I pray to God after then I will just sleep [...] Sometimes if I do remember or want to remember all that has happened to me I will just find a song and sing because if I don´t do that I will start remembering what has happened (18 Years Old Female, Host community).

Meaning-making coping strategies

The ‘thematic meaning-making coping strategies' refers to one of the resource elements that enabled participants to make sense of the displacement. Religious beliefs emerged as a key sub-theme from the thematic meaning-making coping strategies. Majority of participants 13(87%) explained the cause of the Boko-Haram conflict based on their religious beliefs. They actively engaged in searching for a logical explanation and giving meaning to their experiences. Their responses below reveal this:

God loves me [...] I think this displacement is His permissive will [...] and is to show my stand for Him [...] also for me to prove dependence on God in times of crisis (21 Years Old Female, Host Community).

Even though we were displaced, I believe God did not forget us. God loves me even, as God said in the Bible, when you are running from you enemies, He will be with you [...] was it not for God I would not have been able to run from the Boko Haram so I will not forget my God for what He has done for us (17 Years Old Female, Official Camp).

## Discussion

Like other previous research, participants in our study reported experiences of trauma, post-traumatic stress disorder (PTSD) symptoms, transactional sex as a means of income generation and religious coping mechanism. Nevertheless, what this study added is the experiences of the adolescents and their contexts. It addressed their reason for engaging in transactional sex, how it happened, and how they feel about it. All this leads to a better understanding of how to prevent it and how to support people undergoing such. The findings are discussed in line with the identified thematic categories.

### Mental health problems vulnerability

The present study findings are consistent with previous research on IDPs populations. It shows how poor basic infrastructures and overcrowding, food insecurity, and lack of access to safe water, sanitation, and basic health services could increase vulnerability risk capable of affecting mental health and disease trajectories 23–26. The adversity, which the participants faced, reflected a broader vulnerability of their health including mental health problems. Further, this study provides new evidence on adolescent's social change of network. For these participants their loss of parental contacts, family members and friendships were a great concern, as their new social networks in the camp were not supportive. Apparently, adolescents are deemed socially vulnerable when they lacked such essential family support network in time of crisis. They experienced loneliness, due to lack of support in the area of their interpersonal relationship. However, this finding suggests that the social change in IDAs network alongside with their lack of basic needs are among the potential risk factors for developing psychological symptoms.

### Armed conflict experience and their effects

Armed-conflict-displaced populations suffer from both cognitive and emotional reaction symptoms owing to exposure to traumatising events 27. The participants in this study reported several traumatic exposures and not unexpectedly visible psychological symptoms, which included being worried and angry, sadness and sleep difficulties. These findings are congruent with other similar studies of displaced adolescents. For example, Seedat, Nyama, and Njenga 28 reported anxiety, while Garbarino^29^ reported depression, and King'ori, Odera, and Oboka^30^ documented PTSD symptoms. What this qualitative study adds, is the participants experiences of how their homes and communities were destroyed, the dangerous and life-threatening events they encountered, being exposed to killed victims, and loss of family members and friends. Each of these traumatic experiences could cause PTSD symptoms 31 and it is important to understand adolescents' individual and contextualised experiences in order to understand their symptoms and provide adequate support.

### Coping mechanisms

The participants in this study displayed frustration due to their living conditions, and this exacerbated the choice of high-risk survival strategies for the female participants, as they were involved in begging and transacting sex in exchange for needs. Transactional sex can cause serious emotional, psychological, behavioural, and relationship problems such as (depression, suicidal ideation, and substance abuse) or risk the adolescents in contracting sexual transmitted diseases or unwanted pregnancy 32. Of importance, this study uncovered the experiences and the strategies used for survival in a new situation. In addition, being financially poor served as a strong determinant of vulnerability, especially when it requires the female gender within the host community to fix their basic needs, they struggle for money to pay for their education and house rent. In such situations, they are more likely to engage in high-risk survival strategies such as begging and transactional sex.

In terms of religious activities our findings align with Mullica's 33 core psychological dimension of self-healing, in which the individual demonstrates a will to survive and recover by often engaging in religious behavioural approaches such as, prayer, singing of Christian songs and reflecting on the Bible promises. Similarly, Cherewick et al., 34 identified prayer and faith as common coping strategies utilized by adolescents displaced by conflict in Congo. Notably for some participants' engaging into prayer and singing of Christian songs were necessary to suppress their past traumatic memories. This provides insight on extenuating factors underlying why a specific coping strategy was chosen. However, this study finding shows that some IDAs demonstrated resilience in the form of religious activities and meaning making which provided cognitive, emotional as well as problem solving in their adversity.

### Implications and recommendations

Our findings might have practical implications for humanitarian agencies that support IDPs in North-east of Nigeria. Such implications require a suitable support plan that focuses on females IDAs basic needs. Considering that, they are more vulnerable especially to the avoidable high-risk survival strategies (begging and transactional sex). There is need for displaced adolescents' survival means and coping mechanisms to be closely monitored and strengthened with appropriate support and protection services owing to the risks of recourse to high-risk survival behaviour. Along this process, relief from agencies must complement the IDAs' own survival means and coping strategies. It is imperative for NGOs to understand IDAs mental health problems vulnerability and coping strategies from the perspectives of the adolescents and the contexts of their poor living conditions. Moving forward, future studies may focus on establishing links between IDAs high-risk survival means, coping strategies and mental health problems.

### Study limitations

First, we used purposive sampling to recruit IDAs. While this is a commonly used sampling method for qualitative research and is an ethnically safe way in order to minimize both risk of secondary trauma and breaches of participants’ security, there is a risk of sampling bias. Second, the results of this study were generated in a single interview, although this limitation is similar to those that have been well documented for most qualitative research. Considering we selected only a small sample size of (n=15), this makes the findings not generalizable. However, it gathers valuable data and identified themes supported with other similar studies findings. Third, the results of this study are potentially subject to recall bias due to some of the participants were displaced for several years and therefore were less likely to discuss or remember distant experiences that occurred during conflict. Unfortunately, we were unable to interview younger adolescents 10-13 years of age owing to low communication skills. However, those who participated in this study provided important retrospective descriptions of traumatisation events that occurred during various stages of their displacement, which allowed insight into experiences from early adolescence.

## Conclusion

The findings of this study revealed specific contextual mental health problems vulnerability of forcible displaced adolescents in North-east of Nigeria. Profound psychological problems experienced included sadness, worry, angry, uncontrollable thoughts, sleep difficulties, flashback, and nightmares. This study demonstrates that female participants engaged in high-risk survival strategies, which involved begging and transactional sex. These findings suggest that IDAs means of survival need to be closely monitored and strengthened with psychosocial support and protection services owing to the recourse of high-risk survival strategies. Regardless of complex life-changing process for all, some participants demonstrated resilience by engaging in religious behavioural coping such as prayer and singing of Christian songs, which they deem necessary to help suppress their past traumatic memories.
